# Defective CFTR-Dependent CREB Activation Results in Impaired Spermatogenesis and Azoospermia

**DOI:** 10.1371/journal.pone.0019120

**Published:** 2011-05-09

**Authors:** Wen Ming Xu, Jing Chen, Hui Chen, Rui Ying Diao, Kin Lam Fok, Jian Da Dong, Ting Ting Sun, Wen Ying Chen, Mei Kuen Yu, Xiao Hu Zhang, Lai Ling Tsang, Ann Lau, Qi Xian Shi, Qing Hua Shi, Ping Bo Huang, Hsiao Chang Chan

**Affiliations:** 1 Sichuan University-The Chinese University of Hong Kong Joint Laboratory for Reproductive Medicine, West China Institute of Women and Children's Health, West China Second University Hospital, Sichuan University, Chengdu, People's Republic of China; 2 Faculty of Medicine, School of Biomedical Sciences, Epithelial Cell Biology Research Center, The Chinese University of Hong Kong, Hong Kong, People's Republic of China; 3 Shenzhen Key Lab of Male Reproduction and Genetics, Peking University Shenzhen Hospital, Shenzhen, People's Republic of China; 4 Department of Reproductive Physiology, Zhejiang Academy of Medical Sciences, Hangzhou, People's Republic of China; 5 Laboratory of Molecular and Cell Genetics, Hefei National Laboratory for Physical Sciences at Microscale, School of Life Sciences, University of Science and Technology of China, Hefei, People's Republic of China; 6 Department of Biology, Hong Kong University of Science and Technology, Hong Kong, People's Republic of China; Florida International University, United States of America

## Abstract

Cystic fibrosis (CF) is the most common life-limiting recessive genetic disease among Caucasians caused by mutations of the cystic fibrosis transmembrane conductance regulator (CFTR) with over 95% male patients infertile. However, whether CFTR mutations could affect spermatogenesis and result in azoospermia remains an open question. Here we report compromised spermatogenesis, with significantly reduced testicular weight and sperm count, and decreased cAMP-responsive element binding protein (CREB) expression in the testes of CFTR knockout mice. The involvement of CFTR in HCO_3_^−^ transport and the expression of the HCO_3_^−^ sensor, soluble adenylyl cyclase (sAC), are demonstrated for the first time in the primary culture of rat Sertoli cells. Inhibition of CFTR or depletion of HCO_3_^−^ could reduce FSH-stimulated, sAC-dependent cAMP production and phosphorylation of CREB, the key transcription factor in spermatogenesis. Decreased CFTR and CREB expression are also observed in human testes with azoospermia. The present study reveals a previously undefined role of CFTR and sAC in regulating the cAMP-CREB signaling pathway in Sertoli cells, defect of which may result in impaired spermatogenesis and azoospermia. Altered CFTR-sAC-cAMP-CREB functional loop may also underline the pathogenesis of various CF-related diseases.

## Introduction

Cystic fibrosis (CF) is caused by mutations of the cystic fibrosis transmembrane conductance regulator (CFTR), a cAMP-activated anion channel conducting both Cl^−^ and HCO_3_^−^ [1], [2]. A multitude of clinical manifestations are associated with CF, which include chronic lung inflammation/infection, pancreatic insufficiency, intestinal obstruction and infertility/subfertility in both sexes [3], [4], [5]. However, the exact mechanisms underlying various CF-related pathological conditions or diseases remain largely unknown. While previous studies have demonstrated that about 95% of the male CF patients are infertile because of bilateral absence of the vas deferens (CBAVD) [6], whether CFTR mutations may result in other forms of infertility, such as azoospermia, remains an open question.

CFTR expression has been detected in human [7], [8] and rodent [9], [10], [11] testes, both in germ cells and Sertoli cells, suggesting its possible involvement in spermatogenesis. However, controversial data were obtained when CFTR mutation screening was performed in infertile patients with defective testicular function, with some reports indicating the association of CFTR mutations and defects in sperm production [12], [13], [14], [15], but some others rejecting the association [16], [17], [18], [19]. Interestingly, in a study screening a panel of 13 mutations of CFTR in semen specimens from 127 CF-unrelated healthy males attending infertility clinics, fourteen (17.5%) of 80 healthy men with infertility due to reduced sperm quality and two of 21 men (9.5%) with azoospermia carried one CFTR mutation, while no CFTR mutation was detected in 26 males who had normozoospermia [13]. Another study also shows that a higher proportion of 5 T allele, a common variation of the poly(T) in intron 8 of *Cftr*, which has been associated with low level of CFTR expression and with susceptibility to non-classical CF disease patterns, exists in men with severe oligozoospermia [6], [20], suggesting that variation in CFTR may be associated with defects in spermatogenesis. Despite the accumulating evidence indicating possible involvement of CFTR in sperm production, the exact role of CFTR in spermatogenesis has not been elucidated, and thus, the question as to whether CFTR mutations may result in impaired spermatogenesis remains controversial. We undertook the present study to address this question.

## Results

### Impaired spermatogenesis in CF mice models

**Figure 1:**
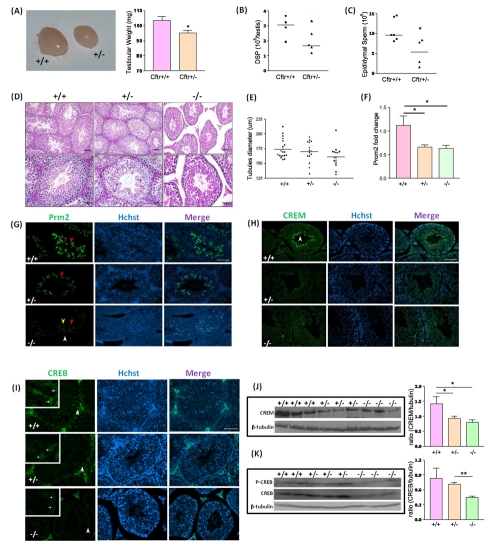
Defective spermatogenesis and altered expression of CREB in CF mice testis. (**A**) Significantly reduced testis size and weight seen in heterozygous S489X mice compared with wild-type mice (n = 3; *:p<0.05). (**B**) Reduced daily sperm production (DSP) values retrieved from heterozygous S489X testes (n = 4; *:p<0.05). (**C**) Reduced sperm numbers recovered from the epididymis of heterozygous S489X mice (+/+ n = 6, +/− n = 4; *:p<0.05). (**D**) H&E staining of the cross sections of wild_−_type, heterozygous and homozygous S489X CF mice testes. Seminiferous tubules remain intact in +/− and −/− CF mice, but looser tubule contacts with larger interstitial space are observed in −/− testes (right) when compared with wild type (left). Leydig cells and peritubular myloid cells are detached from most tubules in −/− testes. Bar  = 20 µm (upper panel) or 50 µm (lower panel). (**E**) Statistic shows slight decrease in tubular diameter is observed in −/− testes. (**F**) Realtime PCR of protamine-2 in S489X CF mice testes. Protamine-2 mRNA level is significantly lowered in heterozygous and homozygous S489X CF mice compared to their wildtype littermates. (**G**) Immunofluorescent staining of Protamine-2 in +/+, +/− and −/− S489X CF mice testes (stage VII–VIII). In +/+ testes, Protamine-2 positive signals are located in elongated spermatids (ES) with strong immunoreactivity (red arrow). In +/− testes, Protamine-2 is found in ES with moderate immunoreactivity (red arrow). In −/− testes, there is weak intensity in the pachytene spermatocytes (P) (white arrow), ES (red arrow) and round spermatids (RS) (yellow arrow). Bar  = 50 µm. (**H**) Immunofluorescent staining of CREM in S489X CF mice testes (stage VIII–I). In wild-type testes, CREM positive signals are localized in RS (white arrow), with strong immunoreactivity. In +/− and −/−testis, CREM showed weak immunoreactivity. Bar  = 50 µm. (**I**) Immunofluorescent staining of CREB in S489X CF mice testes shows stronger immunoactivity in +/+ and +/− mice Sertoli cell compared to that of −/−. Bar = 50 µm. (**J**) Western blot analysis of CREM expression in S489X CF testes. CREM expression is decreased in +/− and −/− testes compared to wild-type (n = 3; *:p<0.05). (**K**) Western blot analysis of CREB expression in S489X CF testes. CREB expression is decreased in −/− testes compared to +/− and wild-type (+/+ n = 2, +/− and −/− n = 3; **:p<0.01). Statistic data are shown as mean±SEM.

To investigate possible involvement of CFTR in spermatogenesis, we used a CFTR knockout (*Cftr^tm1Unc^,* also referred as S489X) mice. Since most homozygous S489X CF mice die at a young age or less frequently available, heterozygous mice were used for quantitative measurement. Morphological study showed that testis tissue size and weight were significantly lower in heterozygous S489X CF mice than that of wild-type control (**Fig. 1A**). Daily sperm production (DSP), which is often used to evaluate the spemiogenesis in the testis [21], was significantly reduced in heterozygous S489X CF mice compared with wide-type control (**Fig. 1B**). The sperm number recovered from the epididymis of the heterozygous S489X CF mice was also significantly lower than that of wide-type control (**Fig. 1C**). H&E staining showed slight decrease in diameter of seminiferous tubules (**Fig. 1D,E**) and slight cytoplasmic shrinkage of spermatocytes and round spermatids (**Fig. S1**) in S489X CF mice. Realtime PCR results showed significant reduction of Protamine-2, a specific marker of spermatid and spermatozoa, in heterozygous and homozygous S489X CF mice at mRNA level (**Fig. 1F**). Immunofluorescence results further demonstrated the down-regulation of Protamine-2 in S489X CF mice at protein level (**Fig. 1G**). CREM, a spermatid-specific transcription factor, was also down-regulated in the homozygous S489X CF mice compared to wild-type as indicated by Western blot and immunofluorescent staining respectively (**Fig. 1H&J**), suggesting defect in spermatogenesis in CF mice. Interestingly, immunofluorescence result showed that cAMP-responsive element binding protein (CREB), an important transcription factor in Sertoli cells during spermatogenesis, was decreased in the Sertoli cells of homozygous S489X CF mice, as compared to heterozygotes and wild-type (**Fig. 1I**). Western blot result also showed downregulation of phosphorylated and total CREB in homozygous S489X CF mice compared to wild-type and heterozygotes (**Fig.1K**). Thus all the spermatogenesis parameters examined indicate that spermatogenesis is compromised in CF mice, confirming a role of CFTR in spermatogenesis. Further more, down-regulation of phosphorylated and total CREB in CF mice suggests that defect of CFTR may lead to abnormal regulation of cAMP-CREB pathway in Sertoli cells, which might be the molecular basis for the spermatogenesis defect observed in CF.

### CFTR is expressed in rodent Sertoli cells and involved in HCO_3_^−^ transport

**Figure 2:**
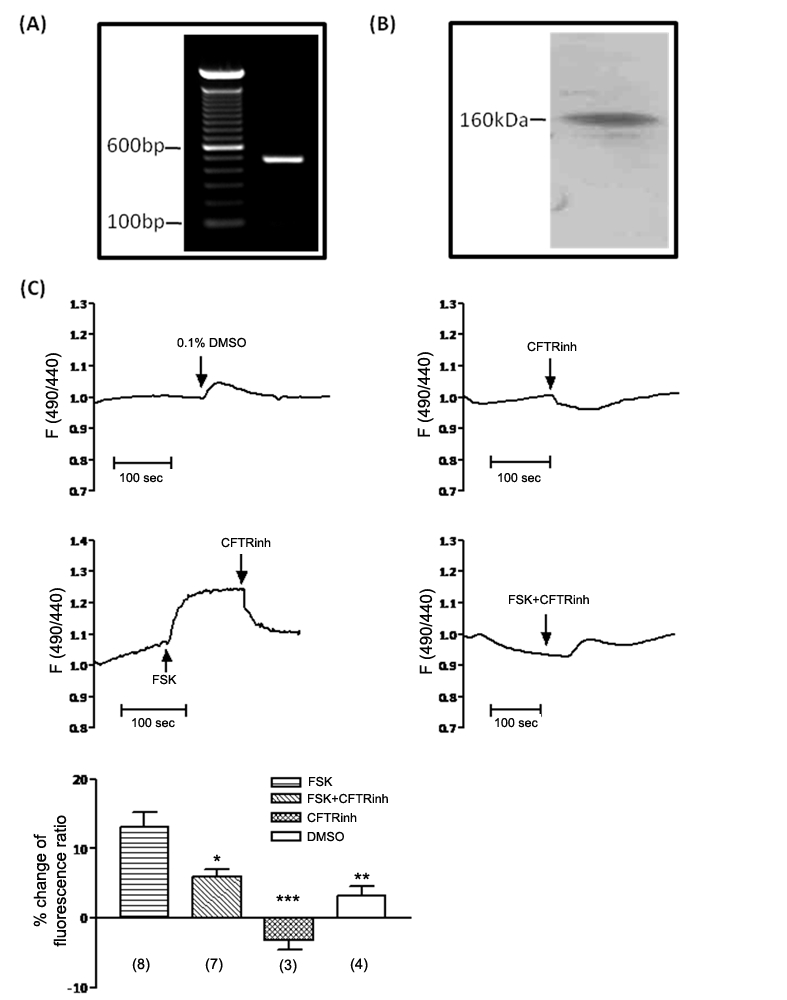
Functional expression of CFTR in rat Sertoli cell primary culture. (**A**) RT-PCR shows CFTR mRNA expression in rat Sertoli cells (expected PCR product size: 481 bp). (**B**) Western blot detects a band at around 160 kDa, showing CFTR protein expression in rat Sertoli cells. (**C**) Examination of CFTR-involved HCO_3_^−^ transport by intracellular pH (pHi) measurement. 10 µM CFTRinh172 results in a slight acidification of pHi at basal condition. 10 µM forskolin induces a rapid alkalization in pHi, which can be reversed by following addition of CFTRinh172. Simultaneous forskolin and CFTRinh172 treatment significantly inhibits forskolin-induced alkalization. (DMSO n = 4, CFTRinh172 n = 3, Forskolin n = 8, CFTRinh172+Forskolin n = 7; *: p<0.05, **:p<0.01,***: p<0.001, compared to Forskolin). Statistic data are shown as mean±SEM.

Hormonal regulation of spermatogenesis, such as by FSH and testosterone, is primarily targeting Sertoli cells, which are in close contact with the germ cells and provide paracrine factors that are important for germ cell development [22]. Given the essential role of Sertoli cells in spermatogenesis and the reported expression of CFTR in these cells [10], we hypothesized that CFTR may play an important role in spermatogenesis by regulating Sertoli cell function. In order to test this hypothesis, we established a primary culture of rat Sertoli cells with >95% purity [23], since primary culture of mouse Sertoli cell is not well established. The expression of CFTR in the cultured Sertoli cells was further confirmed by RT-PCR (**Fig. 2A**) and Western blot (**Fig. 2B**).

A growing body of evidence has demonstrated that CFTR is involved, directly or indirectly, in the transport of HCO_3_^−^, defects of which could be one of the major underlying mechanisms for CF-related clinical presentations [24]. To investigate whether CFTR is involved in HCO_3_^−^ transport in Sertoli cells, we measured intracellular pH using a pH-sensitive fluorescent probe BCECF-AM. The result showed that application of CFTR specific inhibitor CFTRinh172 led to a slight acidification of the cells (**Fig. 2C**). In contrast, application of forskolin, which is known to activate CFTR, could induce alkalization of Sertoli cells, which could be reversed by following addition of CFTRinh172 (**Fig. 2C**). Simultaneous CFTRinh172 and forskolin treatment significantly inhibited forskolin-induced alkalization (**Fig. 2C**). Thus, these results suggest that CFTR may be involved in HCO_3_^−^ transport in the Sertoli cells.

### Involvement of CFTR-mediated HCO_3_^−^ transport in FSH-stimulated cAMP production

Does the CFTR-mediated HCO_3_^−^ transport play any role in spermatogenesis? Follicle-stimulating hormone (FSH) is well-known to regulate the cAMP – CREB pathway in Sertoli cells, which is important for spermatogenesis, through its G-protein-coupled receptor that activates the membrane-bound adenylyl cyclase and elevate intracellular cAMP level [25]. Interestingly, our previous study on sperm has demonstrated that CFTR-mediated HCO_3_^−^ transport can increase intracellular cAMP by activating the soluble adenylyl cyclase (sAC) [26], a proven cellular sensor of HCO_3_^−^ [27], [28]. Thus, the CFTR-dependent and HCO_3_^−^- activated sAC pathway might be an alternative way to increase cAMP in Sertoli cells to regulate spermatogenesis, provided sAC is also expressed in Sertoli cells.

**Figure 3:**
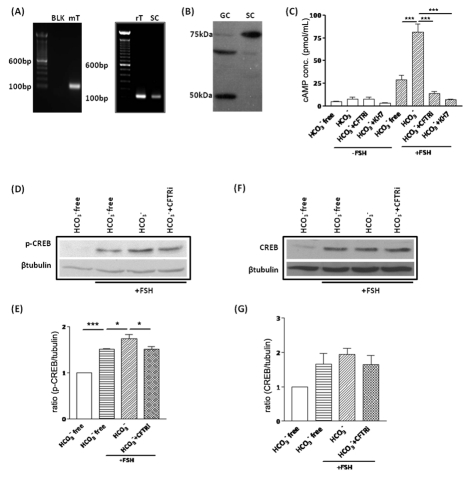
Involvement of CFTR-mediated HCO_3_^−^ transport in FSH-stimulated cAMP production and CREB regulation. (**A**) RT-PCR result shows the expression of sAC in mouse testis (mT), rat testis (rT) and primary Sertoli cells (SC) (expected PCR product size: 119 bp). (**B**) Western blot shows the expression of a 75 kD isoform of sAC in the Sertoli cells (SC). Adult rat germ cell (GC) sample shows predominatly exression of 50 kD, with another unidentified band. (**C**) Effect of HCO_3_^−^, CFTR and sAC inhibitors on 50 ng FSH-stimulated cAMP production, with presence of 25 mM HCO_3_^−^ potentiating FSH-stimulated cAMP production in Sertoli cells, which is abolished by 10 µM CFTRinh172 and 10 µM sAC inhibitor KH7. (n = 3; ***:p<0.01). (**D-E**) Effect of HCO_3_^−^ and CFTR inhibitors on FSH-stimulated CREB phosphorylation. Western blot results shows that 25 mM HCO_3_^−^ potentiates 50 ng FSH-stimulated CREB phosphorylation in Sertoli cells, while 10 µM CFTRinh172 attenuates the effect of HCO_3_^−^ (n = 3; *:p<0.05; ***:p<0.001). (**F-G**) Effect of HCO_3_^−^ and CFTR inhibitor on CREB expression. Western blot results shows that removal of HCO_3_^−^ or treatment with CFTRinh172 slightly decrease total CREB level in the presence of FSH, but without statistic significance (n = 3). Statistic data are shown as mean±SEM. Detailed protocol in Materials and Methods.

Although the critical role of sAC in sperm activation has been demonstrated [29], [30], currently it is still not known whether sAC is expressed in other cell types apart from germ cells in the testis and whether it plays a role in spermatogenesis. RT- PCR result showed a band with expected size of sAC in mouse and rat testis and primary culture of rat Sertoli cells (**Fig. 3A**). Western blot also exhibited a 75 kD sAC isoform expression in Sertoli cells (**Fig. 3B**), which is in accordance with the splicing isoform of sAC reported previously [31].

Intracellular cAMP measurement by ELISA showed that FSH could elevate the intracellular cAMP level in the absence of HCO_3_^−^, but the presence of 25 mM HCO_3_^−^ could significantly enhance the FSH-stimulated cAMP elevation (**Fig. 3C**). The enhancing effect of HCO_3_^−^ was inhibited by both CFTRinh172 and sAC inhibitor KH7 (**Fig. 3C**). These results suggest that CFTR potentiates the FSH-stimulated cAMP production, most probably through the HCO_3_^−^/sAC pathway.

### Involvement of CFTR-mediated HCO_3_^−^ transport in FSH-stimulated CREB phosphorylation and CREB expression

cAMP-responsive element binding protein (CREB) is a well-known target of cAMP involved in spermatogenesis [32], [33]. Coinciding with the result of cAMP assay, HCO_3_^−^ could also enhance the basal and FSH-stimulated CREB phosphorylation in the primary culture of Sertoli cells (**Fig. 3D–E**). The enhancing effect of HCO_3_^−^ on FSH-stimulated CREB phosphorylation could be attenuated by CFTR inhibitor (**Fig. 3D–E**). Similar effect was observed in total CREB level, although without statistic significance (**Fig. 3F–G**). Nevertheless, the simultaneous up-regulation of phosphorylated and total CREB suggests that a positive-feedback loop, similar to that in previous report, may be present in Sertoli cells [32], [33]. Interestingly, a significant percentage of FSH-induced phosphorylation of CREB was independent of HCO_3_^−^ and CFTR as shown in the HCO_3_^−^ free and CFTR inhibitor-treated condition. Since FSH can also activate Ca^2+^-dependent signaling pathways [34], the FSH-induced HCO_3_^−^/CFTR-independent CREB phosphorylation could be contributed by the cAMP-independent intracellular calcium signaling, which has also been demonstrated to increase the phosphorylation of CREB [35]. Nevertheless, bicarbonate-mediated phosphorylation of CREB was abolished by CFTR inhibitor treatment. These results suggest that CFTR-mediated HCO_3_^−^ transport is involved in modulating the FSH-stimulated CREB phosphorylation.

### Decreased CFTR expression and abnormal CREB expression in human azoospermia testes

**Figure 4:**
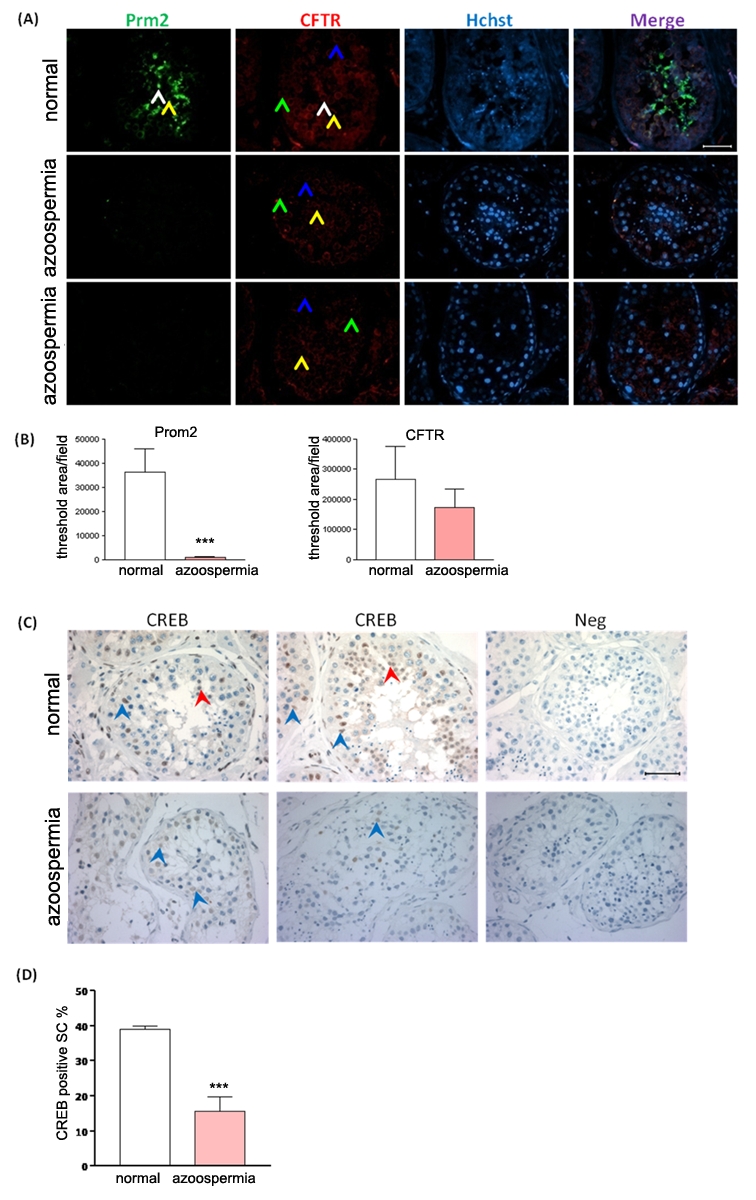
Reduced CFTR and CREB expression in human azoospermia testes. (**A**) Representative immunoinfluoresence staining of CFTR (red) and Protamine-2 (green) in normal human (n = 5) and azoospermia patients (n = 6) testes, counterstained with Hoechst (blue). In the normal human testes, Protamine-2 positive signals are localized in the round spermatids (RS, yellow arow) and elongated spermatids (ES, white arrow) (Stages I–II); CFTR positive signals are localized in pachytene spermatocytes (P, blue arrow), RS and Sertoli cells (SC, green arrow) (stages I–VI), especially in RS. In azoospermia testes, no positive signal of Protamine-2 and weak signals of CFTR are detected (Magnification: 400x). Bar  = 50 µm. (**B**) Statistic analysis of protamine-2 and CFTR positive signal area in normal (n = 5) and azoospermia (n = 6) human testes samples (***:p<0.001). (**C**) Immunohistochemistry of CREB in normal human and azoospermia testes. In normal human testes, CREB positive signals are localized in RS (red arrow) and SC (blue arrow). CREB inmmunoreativity in SC is decreased in the testes with spermatogenic arrest obtained from azoospermia patients. Bar = 50 µm. (**D**) Statistic analysis of CREB positive Sertoli cell percentage in normal (n = 5) and azoospermia (n = 6) human testes (***:p<0.001). Statistic data are shown as mean±SEM.

Both the in vivo and in vitro data indicate that CFTR is involved in spermatogenesis through the activation of cAMP-CREB signaling pathway. To confirm this pathway in human spermatogenesis and test whether abnormality of this pathway may contribute to pathogenesis of azoospermia,we compared the expression profile of CFTR, CREB and the spermatogenic marker protamine-2 in normal human and non-obstructive azoospermia (NOA) testes. The testis sections from azoospermia patients showed that expression of Protamine-2, a spermatid and spermatozoa specific marker used in diagnosis of non-obstructive azoospermia [36], was diminished in azoospermia testes (**Fig. 4A&B**), confirming the defect in spermatogenesis. Immunofluorescence showed that CFTR was expressed in Sertoli cells and germ cells of normal testes. However, its expression was decreased in the testes of azoospermia patients (**Fig. 4A&B**). Consistent with its down-regulation in CF mice and CFTR-inhibited primarily cultured Sertoli cells, CBEB was also decreased in human azoospermia testes (**Fig. 4C&D**), suggesting that the CFTR-dependent CREB pathway may be important for spermatogenesis in human and that defect in this pathway may represent a possible mechanism underlying the pathogenesis of azoospermia.

## Discussion

The present study has demonstrated for the first time the involvement of CFTR in spermatogenesis and elucidated the possible underlying signaling pathway, providing support to the long proposed but intensively disputed link between CFTR mutations and defects in sperm production, such as non-obstructive azoospermia and oligospermia [13], [18], [19], [20], [37], [38]. Apart from the small sample size and inconsistent clinical observations that hampered the claims in previous studies attempting to establish a link between CFTR mutations and abnormality in sperm production, the main reason for the dispute was the lack of a convincing mechanism by which CFTR is involved in the regulation of spermatogenesis. In the present study, we have characterized the spermatogenic phenotypes in CF mice model, which reveals that defects of CFTR cause dysregulation of the major regulatory pathways in spermatogenesis, such as CREB in Sertoli cells and CREM in germ cells, resulting in impaired sperm production. In vitro experiments further demonstrate that CFTR can potentiate the FSH-stimulated CREB phosphorylation by mediating HCO_3_^−^ transport and activation of sAC in Sertoli cells, thus, for the first time, providing a possible mechanism for the involvement of CFTR in spermatogenesis. Since germ cell development critically depends on Sertoli cells, defect in CFTR-dependent FSH-stimulated CREB activation in Sertoli cells could affect germ cell CREM activation and germ cell development as reflected by the diminished expression of a spermatid-specific marker, protamine-2. It should be noted that both CREB and CREM are known to be the master-switch transcription factors coupled to the FSH-regulated cAMP pathway [32], [39], mutations of which are known to be related to male infertility or shown to affect spermatogenesis [40], [41]. The aberrance of both CREB and CREM observed in CF mice testes provides a link between CFTR mutations and defective spermatogenesis. This notion is further supported by the observed down-regulation of CFTR and CREB expression in human azoospermia testes, suggesting that abnormal CFTR-CREB pathway may be a possible cause of azoospermia. The current findings not only reveal a previously undefined role of CFTR in regulation of spermatogenesis but also provide a possible mechanism underlying the pathogenesis of azoospermia in non-CF patients. Of note, there are more than 1800 mutations of CFTR and most of them may not cause typical CF phenotypes, but may lead to compromised spermatogenesis. Therefore, the current findings call for more clinical studies of CFTR mutations screening, both classic genetic mutations and other variant tracts such as IVS8-Tn locus, in azoospermia and severe oligozoospermia patients. In this regard, the rapid development of drug targeting CFTR may provide opportunity for the possible therapy of azoospermia caused by functional deficiency of CFTR in the testis.

Another novel finding of the present study is the expression of sAC in Sertoli cells and its involvement in the FSH-regulated cAMP cascade. Although FSH is known to play an essential role in regulating spermatogenesis through the cAMP pathway in Sertoli cells [33], [42], the present results find that full activation of the FSH-stimulated cAMP pathway depends on HCO_3_^−^, sAC and CFTR, suggesting that the CFTR-HCO_3_^−^-dependent cAMP pathway is not merely an alternative or redundant pathway. In fact, the CFTR-dependent sAC-activated cAMP pathway may be an important loop in the FSH-regulated cAMP cascade for spermatogenesis since mutation or abnormal expression of CFTR could result in impaired spermatogenesis or azoospermia. An increasing body of evidence obtained from other somatic cells, such as lung epithelial cell line Calu-3, indicates that sAC may also be expressed in particular cell microdomain and nucleus as a more fine-tuned and sensitive way to regulate cAMP production and the down-stream target, CREB [43]. The activation of membrane-bound AC by G protein-coupled receptors, such as FSH receptor, may in turn activate CFTR, a cAMP-activated anion channel, allowing for entry of HCO_3_^−^ and subsequent activation of sAC in other cellular compartments. This may represent a more rapid and economic way for cells to response to extracellular or intracellular stimuli [43], such as FSH and testosterone during spermatogenesis.

The present finding that inhibition of CFTR leads to down-regulated FSH-activated cAMP-CREB signaling pathway has also shed new light on the pathogenesis of other CF-related diseases beyond the reproductive tract, since CREB is a master transcription factor capable of regulating a variety of genes involved in different cellular events, including metabolism, neurotransmission, cell cycle/cell survival, cell growth, signal transduction and transport [35]. Interestingly, a recent study has shown dampened beta-adrenergic receptor-activated cAMP pathways in CF cells [44]. The dampened CFTR-dependent cAMP-CREB signaling pathway in response to hormone stimuli, such as adrenalin and FSH, may lead to impaired hormonal regulation and downstream physiological processes, such as epithelial ion transport and spermatogenesis. This may be responsible, at least in part, for the pathophysiological alterations seen in CF. Considering the broad spectrum of CREB target genes and their involvement in a wide range of physiological processes [35], the defective CFTR-dependent CREB pathway and the resulting abnormal expression of its downstream targets may represent an important mechanism underlying various CF-related diseases, and thus, a major therapeutic target for the diseases, including male infertility.

## Materials and Methods

### Animal and cell culture

*cftr^tm1Unc^* (S489X) mice [45] were from Jackson's laboratory and maintained in LASEC of CUHK. 2–6 months old mutant mice and their wild type littermate as control groups were used.

For the primary Sertoli cell culture, 20-day old male S-D rats were used for Sertoli cells isolation as described previously [23]. Hypotonic treatment was performed 2 days after isolation to lyse residual germ cells, and obtained a purity >95%. All the animals handling protocol was approved by the Animal Research Ethics Committee of the University (Ref. No: 04/025/ERG; CUHK 4360/04 M).

### Human testicular tissue samples

Biopsy materials were taken from patients with a normal karyotype who attended the ART (Assisted Reproduction Techniques) Clinic at Peking University Shenzhen Hospital. Prior to any data collection, the experimental protocol was reviewed and approved by the ethics committee of the hospital (Ref. No: 20090018) and all patients signed informed consent approving the use of their tissues for research purposes.

A total of 11 men aged between 23 and 45 years were included in the study. Samples were collected by Department of Pathology, Peking University Shenzhen Hospital. Patients included had no health problems other than azoospermia. Testicular specimens were obtained from the patients with non-obstructive azoospermia with the open micro-testicular biopsy technique. Informed consents were obtained from all patients prior to participation in the study. The original reports of the pathologists were reviewed. Johnsen's score count was used for histological characterization. Normospermia samples (Johnsen's score = 8) and azoospermia samples (Johnsen's score≤4) were used. The specimens (n = 6) were obtained from the patients orchiectomized for the following diagnoses: cysta dermoides, cystadenoma, and prostate cancer.

### Antibodies

Monoclonal anti-CFTR MAb (CF3) was purchased from Enzo Life Sciences (Farmingdale, NY). Monoclonal rabbit anti-CREB, polyclonal rabbit anti-Phospho-CREB (Ser133) was purchased from Cell Signaling Technology (Beverly, MA). polyclonal goat anti-CREM (C-12), polyclonal goat anti-Protamine 2 (C-14) was purchased from Santa Cruz biotechnology (Santa Cruz, CA). Polyclonal rabbit anti-sAC antibody was a gift from Dr. Ping Bo Huang.

### Immunohistochemistry

Clinical tissue specimens and CF mice testis specimens were fixed by formalin, embedded in paraffin and cut into 3-µm sections. Paraffin sections were dewaxed in xylene and rehydrated in descending concentrations of alcohol. Antigen retrieval was achieved by incubation in sub-boil citrate buffer (pH 6.0). All slides were incubated with 3% H_2_O_2_ for 10–15 minutes to block the endogenous peroxidase. After slides were washed with PBS, nonspecific background staining was blocked by 5% normal goat serum (Santa Cruz) for 30 min, followed by overnight incubation at 4°C with anti-CREB (1∶200). For negative control, primary antibodies were omitted. UltraVision ONE Detection System HRP Polymer & DAB Plus Chromogen (Thermo Fisher Scientific Inc.) was used for detection according to the manufacturer's instructions. Mayer's hematoxylin was used for counterstaining.

In quantitative analysis of CREB expression in azoospermia sample, the percentage of CREB-positive Sertoli cells in ten random fields were compared for each sample. Data from 5 normal human and 6 azoospermia patients were used for statistic.

### Immunofluorescence staining

Paraffin sections including clinical tissue specimens and CF mice testis specimens were treated as above. Then the sections were incubated with appropriate diluted primary antibody (anti-CFTR 1∶200; anti-CREM 1∶200; anti-Protamine-2 1∶200; anti-CREB 1∶200) at 4°C overnight, washed with PBS, then incubated with secondary antibody (Alexa 568-conjugated goat-anti-mouse IgG for CFTR, Alexa 488-conjugated rabbit-anti-goat IgG for CREM and Protamine-2, Alexa 488-conjugated goat-anti-rabbit for CREB, from Molecular Probes, Eugene, OR) at 1∶500 dilution in PBS for 1 hour at room temperature. After washed with PBS, the sections were counterstained with Hoechst 33258 for 20 min and covered with Prolong® Gold Antifade Reagent (Invitrogen, Camarillo CA).

Quantitative analysis of human azoospermia samples was performed by Metamorph software, with average optical density of ten random fields were compared for each sample. Data from 5 normal human and 6 azoospermia patients were used for statistic.

### Western blot

Protein was extracted by RIPA cell lysis buffer. 40∼100 µg proteins were resolved by SDS-PAGE followed by transferring onto nitrocellulose membranes. Membranes were blocked with 4% milk in TBS containing 0.05% Tween-20 (TBS-T) for 1 hour, and then incubated in primary antibodies (anti-CFTR 1∶500, anti sAC 1∶500, anti-CREB 1∶200, anti-phospho-CREB 1∶200, anti-CREM 1∶200) in 2% milk at 4°C overnight. Membranes are washed three times in TBS-T followed by incubation with anti-mouse IgG-HRP (1∶10000), anti-rabbit IgG-HRP (1∶10000) or donkey anti-goat IgG-HRP (1∶5000) in 2% milk for 1 hour at room temperature. Following three washes in TBS-T, proteins are detected using an enhanced chemiluminescence kit according to the manufacturer's instructions.

### Intracellular pH measurement

Intracellular pH was measured by BCECF fluorescent imaging. 1 µM BCECF-AM (Molecular Probe) was loaded to cells in Krebs–Henseleit (K-H) solution (in mM, 117 NaCl, 4.7 KCl, 2.56 CaCl_2_, 1.2 MgSO_4_, 24.8 NaHCO_3_, 1.2 KH_2_PO_4_, and 11.1 glucose, pH 7.4) at 37°C for 20 min. During measurement, the cells were incubated in Cl^−^- free K-H solution (in mM, 117 Na-gluconate, 4.7 K-gluconate, 1.2 MgSO_4_, 1.2 KH_2_PO_4_, 24.8 NaHCO_3_, 20 Ca-gluconate, and 11.1 glucose, pH 7.4, gassed with 5% CO_2_ and kept at 37°C). The fluorescent dye was alternately excited with two wavelengths (440 nm and 490 nm) and the emission was measured at 510 nm. The ratio of two signals (440 nm∶490 nm) is directly proportional to pH.

### cAMP assay

Sertoli cells grown in 24-well plate were deprived from HCO_3_^−^ by treatment with HCO_3_^−^-free DMEM/F12 medium for 2 hr. Then the medium was change to HCO_3_^−^-free or 25 mM HCO_3_^−^ DMEM/F12 and incubated for 15 min. In some experiment groups, 10 µM CFTRinh172 (Sigma), 10 µM KH7 (ChemDiv), 50 ng recombinant FSH were added to the media. 100 µM IBMX (Sigma) was supplemented to all media to inhibit the cAMP degradation. After treatment, the cells were lyzed with 0.1 M HCl and centrifuged at 1000 g for 10 min to remove the cell debris. cAMP assay was performed with the cell lysate by enzyme immunoassay following the EIA kit (Assay Design, 901–066) manufacturer's instruction.

### CREB phosphorylation assay

Sertoli cells were deprived from HCO_3_^−^ by treatment with HCO_3_^−^-free DMEM/F12 medium for 2 hr. Then the medium was change to HCO_3_^−^-free or 25 mM HCO_3_^−^ DMEM/F12 and incubated for 30 min. In some experiment groups, 10 µM CFTRinh172 (Sigma) or/and 50 ng recombinant FSH were added to the media. Whole cell protein was extracted by RIPA buffer with protease inhibitor cocktail and sodium orthovanadate. CREB and phospho-CREB was analyzed by Western blot.

### Statistics

For two groups of data, two-tail unpaired Student's t tests were used. For three or more groups, data were analyzed by one-way ANOVA and Tukey's post hoc test. A probability of P <0.05 was considered to be statistically significant.

## Supporting Information

Figure S1 **Enlarged image of spermatocytes and round spermatids of CF mice.** Decreased size of spermatocytes and round spermatids in stage V–VI is observed in −/− testis compared to +/+ and +/−. The cytoplasmic area of spermatocytes and round spermatids shrink progressively from +/− to −/− testes. P: pachytene spermatocytes, RS: round spermatids.
(TIF)
