# Supplementary figures and images for: Defective CFTR-Dependent CREB Activation Results in Impaired Spermatogenesis and Azoospermia
Source: PLoS One. 2011 May 9;6(5):e19120. doi: 10.1371/journal.pone.0019120 (PMC3090391; doi:10.1371/journal.pone.0019120)

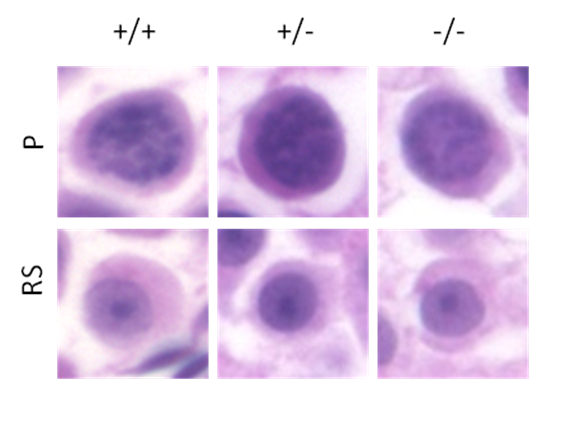

Supplement: Figure S1 — Enlarged image of spermatocytes and round spermatids of CF mice. Decreased size of spermatocytes and round spermatids in stage V–VI is observed in −/− testis compared to +/+ and +/−. The cytoplasmic area of spermatocytes and round spermatids shrink progressively from +/− to −/− testes. P: pachytene spermatocytes, RS: round spermatids. (TIF) [file pone.0019120.s001.tif]
